# Reverse vaccinology for development of vaccine against *Aeromonas hydrophila* infection in nile tilapia *(Oreochromis niloticus)*

**DOI:** 10.1007/s42770-026-02127-z

**Published:** 2026-10-22

**Authors:** Germana Karla de Lima Carvalho, Leticia Stheffany de Barros Cunha, Maria Luiza Evangelista de Brito, Ligia Carolina da Silva Prado, Siomar de Castro Soares, Vasco Ariston de Carvalho Azevedo, Bertram Brenig, Mateus Matiuzzi da Costa, João José de Simoni Gouveia, Gisele Veneroni Gouveia

**Affiliations:** 1 Center for Open Access Genomic Analysis - CALAnGO, Universidade Federal do Vale do São Francisco (Univasf), Rodovia BR-407, CEP 54.300-300, Petrolina, Pernambuco Brazil; 2 Programa de Pós Graduação em Ciências Veterinárias no Semiárido. Campus de ciências agrárias, Universidade Federal do Vale do São Francisco, Rodovia BR-407, KM 12 Lote 543 S/n Projeto de Irrigação Nilo Coelho, Petrolina, Pernambuco Brazil; 3 Programa de Pós Graduação em Ciência Animal. Campus de ciências agrárias, Universidade Federal do Vale do São Francisco (Univasf), Rodovia BR-407, KM 12 Lote 543 S/n Projeto de Irrigação Nilo Coelho, Petrolina, Pernambuco Brazil; 4 Institute of Biological Sciences, Universidade Federal de Minas Gerais, Belo Horizonte, Brazil; 5 Microbiology and Parasitology, Institute of Biological Sciences and Natural Sciences, Universidade Federal do Triângulo Mineiro, Uberaba, Brazil; 6 Universidade Federal da Bahia, Salvador, BA CEP 40170-110 Brazil; 7 Dept. of Molecular Biology of Livestock, Georg August University Göttingen, Burckhardtweg 2, Göttingen, 37077 Germany; 8 Federal Institute (Instituto Federal – IF), Maria Luzia de Araújo Gomes Cabral, 791 - João de Deus, Petrolina, PE CEP 56309-680 Brazil

**Keywords:** Reverse vaccinology, *Aeromonas hydrophila*, Nile tilapia, Vaccines targets

## Abstract

*Aeromonas hydrophila* is one of the main pathogens of Tilapia and has a wide range phenotype, making it difficult to prevent by a unique vaccine. And turning the majority of the treatment using antibiotics. The aim of this work was to predict vaccine candidate with potential efficacy to protect *Oreochromis niloticus* against *Aeromonas hydrophila*, applying bioinformatics tools in a reverse vaccinology approach. It was obtained 35 *A. hydrophila* genomes in the São Francisco Valley in Petrolina/PE/Brazil, as well as 24 *A. hydrophila* and 02 *O. niloticus* complete genomes available in Genbank. After identification of core genes of *Aeromonas hydrophila* and a subtractive genomics analysis between the *O. niloticus* genome and *A. hydrophila* genome, 32 of the 178 resulting proteins, were considered likely vaccine targets as they showed a high adherence probability (> 0.51) in the Vaxign tool. After BLAST analysis against the database of essential genes (DEG), 18 proteins showed defined criteria e-value (≤ 10^− 6^) and a relevant similarity with the proteins of pathogenic species from the genus *Aeromonas*. Were selected 10 proteins, based on the criteria of portability adhesion, molecular mass between 19 and 80 kDa and presence of one or no transmembrane helix. The analyses identified nine proteins [secreted (*n* = 8) and PSE (*n* = 1)] with immunological and novel properties as candidates for vaccine targets. Finally, *in vitro/in vivo tests will be necessary* to experimentally validate the results of this search.

**Supplementary Information:**

The online version contains supplementary material available at https://doi.org/10.1007/s42770-026-02127-z.

## Introduction

Aquaculture contributes to global socioeconomic development, constituting an important source of income for small-scale producers, and also a market for large multinational companies [1]. Aquaculture growth is predicted to increase in the next years, even in a pessimistic scenario, showing the importance for food security [2]. *Oreochromis niloticus* is the third most produced species in aquaculture worldwide [3], and is an important product in Brazil, which produced 499,364,278 kilos of Nile tilapia in 2024 [4].

The culture system for aquatic animals, including the Nile Tilapia, uses high stocking density, and it is successful economically. However, the high stock densities may cause stress, food and shelter competition, leading to a decrease in animal growth, besides the possibility of a disease outbreak [5, 6] which could lead to the development of infectious diseases in fish. Among bacterial infectious diseases, *Aeromonas hydrophila* is one of the most important pathogens and causes millions of dollars in economic losses in the global aquaculture industry [7, 8]. *Aeromonas hydrophila* infection can present visible symptoms such as loss of scales, tail erosions, skin redness and ulceration, exophthalmia, cloudiness of the eyes, abdominal distention and erratic movements. And internal symptoms such as congestion in the abdominal area, liver paleness, congested spleen, septicemia of the intestine and enlargement of the internal organs [9, 10].

Currently, most diseases caused by bacteria in fish are treated by the use of antibiotics. The administration of these drugs to combat infections can input those chemicals into the water. These effects tend to be higher if water renewal is not carried out properly, putting food security at risk by the presence of antibiotic residues in fish [11, 12]. Antibiotics on the water surface, even at sub-inhibitory concentrations, can lead to the emergence of resistance, both in commensal bacteria in the human gut and in fish and aquatic bacteria, with possible spread of resistance genes in different bacterial populations. The accumulation of these substances in sources of human consumption, can be a risk to human health [11, 13]. The presence of antibiotics in the surface water also poses a risk to the biodiversity, aquatic organisms and fish immunity by causing changes in the microbiomes of fishes, changing the susceptibility to pathogens [13, 14].

Therefore, other strategies are needed to minimize the economic, environmental and public health impacts arising from the occurrence of bacterial infections in fish production. One aspect that has been growing in the market is the search for vaccines, a method that prevents infections caused by pathogens and minimizes the use of antibiotics, being a possibility to reduce the cost related to the loss of animals by infection [15, 16].

The first vaccine to immunize tilapia against *A. hydrophila* was produced in 1986 and was a monovalent whole cell vaccine [17]. Since then, some experimental vaccines have shown promise for preventing infection caused by this pathogen in different fish species, mainly in warm water species [18]. However, the effectiveness of a commercial vaccine is hampered by the various phenotypic and serological specificities of the *Aeromonas* spp complex, which is an obstacle that still persists. The ability of *Aeromonas* spp. to persist in the environment is a crucial factor for acquiring genes through horizontal transfer [19, 20].

Another obstacle to the development of commercial vaccines against *A. hydrophila* is the great diversity of pathogen strains, with the complexity of virulence genes, which leads to the failure of the vaccine to protect against heterologous strains [16]. As a result, new technological vaccines are being developed and experimentally tested against *A. hydrophila* in different species of fish, in different regions of the world [21].

Modern techniques for developing immunogens include reverse vaccinology, which aims to understand potential candidates for vaccine production by analyzing the whole bacterial genome. The reverse vaccinology selects the proteins that can act as immunogenic, saving up money and time for researchers. The effectiveness of these compounds increases significantly when there is a complete understanding of the mechanisms of host-pathogen interaction [18, 21]. Therefore, the aim of the present study was to identify in silico targets for a vaccine against *A. hydrophila* in the Nile tilapia.

## Materials and methods

### Genomes

Thirty-five *A. hydrophila isolates* were sequenced using an Illumina^®^ HiSeq in the Federal University of Minas Gerais. The isolates were obtained in the region of São Francisco Valley in Petrolina/PE/Brazil, from the kidney of healthy Nile tilapia (*n* = 3) and diseased Nile tilapia (*n* = 7), diseased Pirarara (*Phractocephalus hemioliopterus*) (*n* = 1), Pacamãs (*Lophiosilurus alexandri*) patients and “pool” of Branchonetas (*Dendrocephalus brasiliensis*) (*n* = 10) and from collections at different locations (*n* = 14) (Tables 1 and 4 of the Supplementary Material).

Raw genomes were submitted to the pipeline (https://github.com/engbiopct/assembly-hiseq) sequence quality was assessed by FastQC software [22], adapters were removed using AdapterRemoval v.2 [23] and KmerStream [24] was used to estimate the best values of k-mer.

SPAdes [25] and Edena [26] software in conjunction with the Unicycler Assembly Pipeline [27] were used to assemble the genomes. The CD-HIT software [28] was used to reduce the computational cost and facilitate sequence assembly by removing the duplicates from the sequencing process. The assemblies from the different softwares were evaluated with QUAST [29], considering the parameters size (approximately 4.6 MB), N50 and GC content (58%). The Prokka v1.14.6 software was used for identify genes and generates some files as, faa file (Fasta amino acid), fna file (Fasta nucleotide acid), ffn (Fasta functional nucleotide), frn (Fasta RNA nucleotide) as output [30].

In addition to the genomes sequenced in the present study, 24 complete genomes of *A. hydrophila*, available in Genbank (Tables 2 and 5 of the Supplementary Material), accessible on the National Center for Biotechnology Information (NCBI) website (address http://www.ncbi.nlm.nih.gov ) were used.

In reverse vaccinology analyses, two complete genomes of *O. niloticus* were also used, which are also available in NCBI (Tables 3 and 6 of Supplementary Material).

### Core genome identification and subtractive genomics

Complete genomes were converted into FASTA amino acid format and transferred to the OrthoFinder software v1.x ( https://github.com/davidemms/OrthoFinder ) [31]. The genes were then categorized into orthologue groups, and the output give some files, as Orthogroups/Orthogroups_UnassignedGenes.tsv (For the genes classifies the genes that are exclusive for just one strain and Orthogroups/Orthogroups.GeneCount.tsv. Three gene groups were made “in-house scripts”: Core genes (present in all strains analyzed), accessory genes (present in some but not all lineages, representing accessory genome) and singletons (specific genes present in only one lineage) [32]. Only the genes of the core genome were included in the next step.

Also, a BLASTp (https://blast.ncbi.nlm.nih.gov/) of the amino acid sequences (faa) was performed by OrthoFinder with the proteins belonging to the Nile Tilapia genome, and was also used to identify the proteins contained in *A. hydrophila* that are non-homologous to the host, to mitigate the risks of autoimmunity [33]. The result was given on the Phylogenetic_Hierarchy/Orthogroups.tsv file, which gives the results about the blast with the files from the host and the *A. hydrophila* gene orthologue group.

### Prediction of subcellular localization of proteins and identification of essential genes

Subcellular localization prediction was performed using the SurfG+ v1.0 software [34]. The DEG website (*Database of Essential Genes*) (http://origin.tubic.org/deg/public/index.php), contains records of essential genes from bacteria and eukaryotes [35], and was used to verify the significance of each identified protein. To predict vaccine targets, only those proteins considered essential for the bacteria survival were used with the defined criteria e-value (≤ 10^− 6^). Proteins identified as being membrane components, PSE (possibly surface exposed) and secreted proteins were selected for reverse vaccinology analyzes [34].

### Prediction of antigenic properties and selection of vaccine targets

The antigenic properties of the proteins identified as membrane, PSE and secreted components were determined using the Vaxign website (http://www.violinet.org/vaxign/). The exported proteins predicted by SurfG+ v1.0 software were analyzed using Vaxign software, which does include de PSORTb tool [36]. The adhesion capacity of the targets was estimated by SPAAN, a Vaxign tool with sensitivity of 89% and specificity of 100% that evaluated the adhesion probability of the targets, with a determined cut-off of 0.51 [37, 38].

The presence of Signal Peptide was evaluated on the website SignalP 5.0 for gram- and default treshold (http://www.cbs.dtu.dk/services/SignalP/ ) and the number of transmembrane helices from the selected proteins was evaluated on TMHMM 2.0 (http://www.cbs.dtu.dk/services/TMHMM/). The selected targets were evaluated for family, domain, gene and product on the InterPro (Classification of protein families) website (https://www.ebi.ac.uk/interpro/search/sequence-search ).

## Results

### Genomes

The analysis with the OrthoFinder software identified 2,823 genes belonging to the core genome of *A. hydrophila* and the accessory and singletons genes were excluded. From the proteins files 332 proteins from the *Aeromonas hydrophila* are non-homologous to the host *O. niloticus*.

### Core genome identification, subtractive genomics, prediction of subcellular localization of proteins and identification of essential genes

Among the 332 proteins belonging to the core genome of *A. hydrophila* and not homologous to *O. niloticus*, 178 proteins were identified as PSE, secreted or membrane proteins and 154 proteins were predicted to be cytoplasmic (Table 1) and, therefore, were not considered in this study, but could be explored as possible drug targets in future studies.

**Table 1:** Location of protein targets

Cell location	Number of proteins found
Cytoplasmic	154
PSE	34
secreted	42
Membrane	102
**Total**	**332**

PSE - Surface-Exposed Proteins

### Prediction of antigenic properties and selection of vaccine targets

Of the 178 PSE, secreted or membrane proteins, 32 were considered by Vaxign as probable immunogenic targets as they had a high adhesion probability (> 0.51) (Tables 7 and 8 in Supplementary Material). Out of 32 proteins, 18 were considered essential in the DEG database [24] (Table 9 in Supplementary Material).

In the group of the 18 essential proteins, the 10 proteins with the highest adhesin probability identified by Vaxign were selected. One aspect for the protein’s selection was the number of transmembrane helices, because proteins with a higher number o transmembrane helices are difficult to synthetize and purify the protein in the future study with these proteins. From the 10 proteins, WP_219313282.1; WP_113995956.1; WP_219312629.1; WP_029305116.1 were classified as secreted and outer membrane, and it is probably because these proteins are secreted to reach the outer membrane. The protein WP_043160143.1 is classified as cytoplasmatic membrane and membrane, so this it is located on the inner membrane, having lower chances to bind an antibody. The other five proteins were classified as unknow and secreted, so the software did not get a good score, giving the result as unknow.

Considering the 10 proteins analyzed, WP_043160143.1 has no signal peptide, with a molecular mass of less than 19 kDa, in addition to having 4 transmembrane helices. Due to these aspects, this protein was excluded from the study. All the other 9 selected proteins, WP_219313282.1; WP_219311764.1; WP_011705502.1; WP_219311898.1; WP_113995956.1; WP_219312142.1; WP_219312629.1; WP_029305116.1 and WP_011707296.1 presented the necessary requirements and fit within the methodological criteria adopted in this study (Table 10 in Supplementary Material). The nine proteins selected will proceed for future studies, filtering for another complementary parameters.

## Discussion

Proteins located in the membrane, PSE or secreted are the first to come into contact with the host organism’s defense, therefore having a greater capacity to produce adaptive immune responses in cellular or humoral responses [39]. As the *A hydrophila* species is a gram-negative bacterium, it does exhibit a thin layer. Proteins with these predictions are more exposed to the extracellular environment, facilitating the recognition of this type of response. Because of this, we considered proteins located near or on the membrane as the main selection parameter [40].

Essentiality refers to genes that are essential for maintaining the basic functions required to sustain the cellular life of an organism [35]. There is a consensus on the essentiality of genes belonging to the functional categories related to the processing of genetic information (DNA replication, transcription and translation). For reverse vaccinology, this part of the approach aims to identify targets that are conserved in different organisms and therefore constitutively expressed, leading to a greater probability of eliciting an immune response. Therefore, the prediction identification of possible essential proteins, within the set evaluated, constituted an important step in reverse vaccinology, which preceded the analyzes of the absence or presence of signal peptide, as well as the number of transmembrane helices.

A signal peptide was adopted for the selection of the vaccine’s target proteins, and their possible location was recorded. Furthermore, proteins with high vaccine potential were considered to be those with 1 (one) or no transmembrane helix (TMHMM) and a molecular mass below 100 kDa (Table 9 in Suppplementary Material). And the presence of a signal peptide implies that the protein may not be cytoplasmatic [41].

Signal peptide is a short peptide, usually 16–30 amino acid residues in length [42], present at the N-terminus or occasionally the C-terminus [43] of most newly synthesized proteins that are destined to the secretory pathway [44]. In prokaryotes, they direct the newly synthesized protein to the SecYEG protein channel located in the plasma membrane. Therefore, the efficiency of protein secretion is strongly determined by the signal peptide [45, 46].

The transmembrane helices cross the outer membrane several times, forming loops where epitopes are exposed and amino acids are organized with precision, enabling contact with the immune system [47]. However, they constitute a limiting factor for in vitro protein synthesis, a great number of transmembrane helices turn the recombinant proteins difficult to express [48]. For this reason, proteins that present a set of characteristics that facilitate the *in vitro preparation*, i.e. proteins with few transmembrane helices, were prioritized in this study.

The selection of proteins, based on the criterion of apparent molecular mass, followed the same logic of facilitating vaccine production. Molecular masses of proteins for expression on *Escherichia coli* must have an appropriate size for the bacteria machinery. A size between 600 and 800 amino acids decreases the chances of being soluble after expression. While proteins below the size of 100 amino acids have a better proportion of soluble proteins after expression, still has a decreased soluble expressed protein. The range of 100 to 600 amino acids permits an easy extraction of soluble proteins when compared with the sizes indicated above [49–51].

Above all, the use of reverse vaccinology for the development of a vaccine used in fish farming is new, considering the last ten years in the literature. Since the 1980s, there have been promising experimental vaccines to protect various fish species against the pathogen *A. hydrophila*. However, due to the great genetic variability between the heterologous strains of this bacterium, the conventional bacterial vaccines already available have major limitations in their coverage effectiveness.

The protein Carotenoid 1,2-hydratase (WP_219313282) does not have many descriptions on literature, especially connected with *Aeromonas*, however, the structure reveals some functions based on structural signature. The WP_219313282 protein belongs to the protein family PF09410 and presents a lipocalin-like fold and a calycin signature, and it is suggested based on sequence homology that this protein is associated with survival of the bacteria under oxidative stress [52, 53]. Another characterization is based on a generic annotation and gets a name of carotenoid 1,2-hydratase [*Aeromonas* sp. 2692-1], based on function is described in a study conducted by Hiseni et al. [54], to have the potential to play a role in stabilizing the hydrophobic region or the function of a “molecular trigger” passing signals to the inner membrane.

Metallo-beta-lactamase (WP_219312142.1) is member of the protein family MBL-fold metallohydrolase and catalyzes the hydrolysis of the beta-lactam ring, inactivating the antimicrobial [54, 55] and preventing it from showing activity against the enzymes responsible for the synthesis of the bacterial cell wall [56]. Many Gram-negative bacteria naturally have β-lactamases, that are, these chromosomally mediated enzymes. Several types of these enzymes can be produced by bacteria of the genus *Aeromonas.* Increased resistance to antimicrobials, particularly β-lactams, is a characteristic of the *Aeromonas genus* and has been reported in both clinical and environmental samples [57].

Maltoporin (WP_113995956.1) belongs to a group of proteins (porins) that restrict the access of many antimicrobial agents to the cytoplasm of Gram-negative bacteria, which, in general, are more resistant to drugs than Gram-positive bacteria. In Gram-negative bacteria, the periplasm contains a large number of hydrolytic enzymes, antibiotic inactivating enzymes, chemoreceptors and oligosaccharides, which appear to help the bacteria to resist the osmolarity of the medium [58]. This suggests that maltoporin may be able to reduce the initial activation barrier, due to its affinity site pre-arranged with a hydrophobic environment and a distribution charge complementary to the permeant sugar. This mechanism of transition state stabilization by specific solvation substitution forms the basis of the enzymatic catalysis process in a polar solvent [59]. Therefore, specific studies of the maltoporin role in the development of drugs for the treatment of motile *Aeromonas* septicemia (MAS) must be carried out.

YceI family proteins (WP_011705502.1) are poorly characterized, which makes studies focused on its behavior and functional details even more important. The YceI like domain is a part of the lipocalin superfamily, the proteins belonging to this family are characterized for the lipocalin fold-like. In this family it has been reported some functions as the sequestrations of fatty-acids, involvement in antibiotic resistance and response to different kinds of stress by different species [60, 61]. Different species gets no sequence homology, turning into different functions [62, 63]. Results of this nature may be an indication that other functions that are still unknown and related to the immunogenicity of this family of proteins should be the target of targeted studies.

Cytochrome c-type biogenesis protein CcmE (WP_219311898.1) and YtfJ family protein (WP_011707296.1) have very different apparent molecular masses: 42.90 kDa and 19.61 kDa respectively. Feature that favors in vitro analysis and the purification process of these proteins. Among other aspects, they do not have any transmembrane helix and their adhesin probability by Vaxign were 0.719 and 0.603, respectively.

The proteins YceI family protein (WP_011705502.1), Maltoporin (WP_113995956.1), Vitamin B12 transporter (WP_219312629.1), Lytic transglycosylase (WP_219311764) and TonB-dependent siderophore receptor (WP_029305116.1) are virulence factors, verified by means of accessing PBIT (*Pipeline Builder for Identification of drug targets for infectious diseases*) [64]. The identification of these virulence factors was relevant, as there is a direct correlation between them and the pathogenicity of the bacteria [65]. The tonB-dependent receptors have already been found in other studies, confirming the potential of the TonB-dependent siderophore receptor (WP_029305116.1) as a good target for a vaccine [48, 66, 67].

It is proposed that these five proteins to be the first to be tested at in vitro analysis and in the future *in vivo tests* on the species *O. niloticus*, since the identification of virulence factors is crucial for the development of effective vaccines, as they stimulate a protective response against infections caused by these pathogens [68]. The parameters testes are an initial selection of proteins from *Aeromonas hydrophila* to future analysis, which will need to be performed another in silico test with the resulting proteins from this work.

## Conclusion

The nine proteins selected from reverse vaccinology analysis were considered potential vaccine targets by reverse vaccinology analysis. Therefore, the information listed in this study can support and guide the direction of future research towards the construction of a vaccine against *A. hydrophila.* Therefore, this research can be considered as a starting point for developing a targeted and effective vaccine against *A. hydrophila*, accelerating the in vitro testing stages and minimizing the number of animals needed for in vivo testing. however, these experimental validation steps, are necessary to prove the efficiency of these targets, as well as the stability, safety and immunogenicity of the vaccine to be developed.

## Supplementary Information

Below is the link to the electronic supplementary material.

Supplementary Material 1 (DOCX 72.6 KB)

## Data Availability

The data is available on the NCBI website (https://www.ncbi.nlm.nih.gov/), the GenBank number is shared on the tables in the supplementary material for each genome used for analysis.
