# Supplementary material for: Reverse vaccinology for development of vaccine against Aeromonas hydrophila infection in nile tilapia (Oreochromis niloticus)
Source: Braz J Microbiol. 2026 Oct 22;57(1):300. doi: 10.1007/s42770-026-02127-z (PMC13691019; doi:10.1007/s42770-026-02127-z)
Supplement: Supplementary file 1 [file 42770_2026_2127_MOESM1_ESM.docx]

**REVERSE VACCINOLOGY FOR DEVELOPMENT OF VACCINE AGAINST *Aeromonas hydrophila* INFECTION IN NILE TILAPIA *(Oreochromis niloticus)***

**Authors:** Germana Karla de Lima Carvalho (<https://orcid.org/0009-0009-7337-6192>) ^1,2, 8^;

Leticia Stheffany de Barros Cunha (<https://orcid.org/0000-0003-2501-919X>) ^1,2^;

Maria Luiza Evangelista de Brito ([https://orcid.org/0000-0002-9233-4758)^1,3^](https://orcid.org/0000-0002-9233-4758)1,3);

Ligia Carolina da Silva Prado (0000-0002-4974-8269) ^4^;

Siomar de Castro Soares (<https://orcid.org/0000-0001-7299-3724>) ^5^;

Vasco Ariston de Carvalho Azevedo (<https://orcid.org/0000-0002-4775-2280>) ^6^;

Bertram Brenig (<https://orcid.org/0000-0002-7635-9656>) ^7^;

Mateus Matiuzzi da Costa (<https://orcid.org/0000-0002-9884-2112>) ^1,2,3^;

João José de Simoni Gouveia (<https://orcid.org/0000-0002-0438-094X>) ^1,2,3^;

Gisele Veneroni Gouveia (<https://orcid.org/0000-0003-1074-5711>) ^1,2,3^.

**Affiliations:**

^1^ Center for Open Access Genomic Analysis - CALAnGO, Federal University of vale do São Francisco, Rodovia BR-407, CEP 54.300-300. Petrolina, Pernambuco, Brazil.

^2^ Postgraduate Program in Veterinary Sciences in Semiarid. Agricultural Sciences Campus, Federal University of Vale of São Francisco (Univasf). Rodovia BR-407, KM 12 Lote 543 S/n Projeto de Irrigação Nilo Coelho, Petrolina, Pernambuco, Brazil. CEP 56.300-990.

^3^Postgraduate Program in Animal Science. Agricultural Sciences Campus, Federal University of Vale of São Francisco (Univasf). Rodovia BR-407, KM 12 Lote 543 S/n Projeto de Irrigação Nilo Coelho, Petrolina, Pernambuco, Brazil. CEP 56.300-990.

^4^Institute of Biological Sciences. Federal University of Minas Gerais, Belo Horizonte, Brazil.

^5^ Microbiology and Parasitology, Institute of Biological Sciences and Natural Sciences, Federal University of Triângulo Mineiro, Uberaba, Brazil.

^6^ Federal University of Bahia, Salvador - BA, Brazil. CEP 40170-110.

^7^ Dept. of Molecular Biology of Livestock, Georg August University Göttingen, Burckhardtweg 2, 37077 Göttingen, Germany.

^8^ Federal Institute (Instituto Federal – IF). Street: Maria Luzia de Araújo Gomes Cabral, 791 - João de Deus, Petrolina - PE, Brazil. CEP 56309-680.

**Corresponding author:** Gisele Veneroni Gouveia

Email: [gisele.veneroni@univasf.edu.br](mailto:gisele.veneroni@univasf.edu.br)

**Supplementary Material**

**Table 1.** Correspondence of sample and host from which the sample was collected.

| **GenBank No.** | **Host** | **Isolation source** |
| --- | --- | --- |
| GCA_017310195.1 | *Dendrocephalus brasiliensis* | ------- |
| GCA_019348925.1 | *Oreochromis niloticus* | Kidney |
| GCA_022700855.1 | *Dendrocephalus brasiliensis* | ------- |
| GCA_019399935.1 | *Dendrocephalus brasiliensis* | ------- |
| GCA_019399965.1 | *Dendrocephalus brasiliensis* | ------- |
| GCA_022700815.1 | *Dendrocephalus brasiliensis* | ------- |
| GCA_017310135.1 | *Lophiosilurus alexandri* | Kidney |
| GCA_017310075.1 | *Lophiosilurus alexandri* | Kidney |
| GCA_018094775.1 | *Lophiosilurus alexandri* | Kidney |
| GCA_019348795.1 | *Oreochromis niloticus* | Kidney |
| GCA_019348875.1 | *Oreochromis niloticus* | Kidney |
| GCA_038524185.1 | *Oreochromis niloticus* | Kidney injury |
| GCA_018094725.1 | *Oreochromis niloticus* | Kidney |
| GCA_021735805.1 | *Oreochromis niloticus* | Kidney |
| GCA_053543895.1 | *Oreochromis niloticus* | Kidney injury |
| GCA_053543875.1 | *Oreochromis niloticus* | Kidney injury |
| GCA_053543855.1 | *Oreochromis niloticus* | Kidney injury |
| GCA_019348745.1 | *Oreochromis niloticus* | Kidney |
| GCA_053346735.1 | *Oreochromis niloticus* | Kidney injury |
| GCA_019348935.1 | *Oreochromis niloticus* | Kidney |
| GCA_021735825.1 | *Oreochromis niloticus* | Tissue sample |
| GCA_019348915.1 | *Oreochromis niloticus* | Kidney |
| GCA_016805405.1 | *Oreochromis niloticus* | Kidney |
| GCA_019348835.1 | *Oreochromis niloticus* | Kidney |
| GCA_017310155.1 | *Oreochromis niloticus* | Kidney |
| GCA_018094805.1 | *Oreochromis niloticus* | Kidney |
| GCA_017310215.1 | *Oreochromis niloticus* | Kidney |
| GCA_017310115.1 | *Oreochromis niloticus* | Kidney |
| GCA_019348895.1 | *Oreochromis niloticus* | Kidney |
| GCA_019348855.1 | Oreochromis niloticus | Kidney |
| GCA_019399995.1 | Phractocephalus hemioliopterus | Kidney |

| **GenBank No.** | **Host** | **Isolation source** |
| --- | --- | --- |
| GCA_017310195.1 | Dendrocephalus brasiliensis | ------- |
| GCA_019348925.1 | Oreochromis niloticus | Kidney |
| GCA_022700855.1 | Dendrocephalus brasiliensis | ------- |
| GCA_019399935.1 | Dendrocephalus brasiliensis | ------- |
| GCA_019399965.1 | Dendrocephalus brasiliensis | ------- |
| GCA_022700815.1 | Dendrocephalus brasiliensis | ------- |
| GCA_017310135.1 | Lophiosilurus alexandri | Kidney |
| GCA_017310075.1 | Lophiosilurus alexandri | Kidney |
| GCA_018094775.1 | Lophiosilurus alexandri | Kidney |
| GCA_019348795.1 | Oreochromis niloticus | Kidney |
| GCA_019348875.1 | Oreochromis niloticus | Kidney |
| GCA_038524185.1 | Oreochromis niloticus | Kidney injury |
| GCA_018094725.1 | Oreochromis niloticus | Kidney |
| GCA_021735805.1 | Oreochromis niloticus | Kidney |
| GCA_053543895.1 | Oreochromis niloticus | Kidney injury |
| GCA_053543875.1 | Oreochromis niloticus | Kidney injury |
| GCA_053543855.1 | Oreochromis niloticus | Kidney injury |
| GCA_019348745.1 | Oreochromis niloticus | Kidney |
| GCA_053346735.1 | Oreochromis niloticus | Kidney injury |
| GCA_019348935.1 | Oreochromis niloticus | Kidney |
| GCA_021735825.1 | Oreochromis niloticus | Tissue sample |
| GCA_019348915.1 | Oreochromis niloticus | Kidney |
| GCA_016805405.1 | Oreochromis niloticus | Kidney |
| GCA_019348835.1 | Oreochromis niloticus | Kidney |
| GCA_017310155.1 | Oreochromis niloticus | Kidney |
| GCA_018094805.1 | Oreochromis niloticus | Kidney |
| GCA_017310215.1 | Oreochromis niloticus | Kidney |
| GCA_017310115.1 | Oreochromis niloticus | Kidney |
| GCA_019348895.1 | Oreochromis niloticus | Kidney |
| GCA_019348855.1 | Oreochromis niloticus | Kidney |
| GCA_019399995.1 | Phractocephalus hemioliopterus | Kidney |

**Table 2.** Identification of 24 complete *Aeromonas hydrophila* sequences accessed by NCBI to vaccinology analysis.

| **Reference Sequence** | **Submitted Sequence** | **Strain** | **Origin** |
| --- | --- | --- | --- |
| GCF_000940915.1 | GCA_000940915.1 | AL06-06 | Auburn University Southeastern Cooperative Fish Disease Laboratory in Greensboro, Alabama |
| GCF_000963645.1 | GCA_000963645.1 | AH10 | National Aquatic Pathogen Collection Center, Shanghai, China |
| GCF_001455365.1 | GCA_001455365.1 | JBN2301 | Wuming Yang, Wuhan Polytechnic University, Wuhan, China |
| GCF_001518775.1 | GCA_001518775.1 | D4 | College of Fisheries, Huazhong Agricultural University, Wuhan, PR China |
| GCF_001683535.1 | GCA_001683535.1 | GYK1 | Pearl River Fisheries Research Institute of Chinese Academy of Fishery Sciences, Liwan District, Guangzhou, Guangdong Province. |
| GCF_001687125.1 | GCA_001687125.1 | AHNIH1 | NHGRI/NIH  Perirectal swab, *Homo sapiens* |
| GCF_001895965.1 | GCA_001895965.1 | MX16A | Fisheries College, Jimei University, Xiamen, Fujian - China |
| GCF_003014775.1 | GCA_003014775.1 | KN-Mc-1R2 | Korea Research Institute of Bioscience and Biotechnology |
| GCF_003491225.1 | GCA_003491225.1 | ZYAH72 | HuaZhong Agricultural University |
| GCF_003491245.1 | GCA_003491245.1 | ZYAH75 | HuaZhong Agricultural University |
| GCF_004296435.1 | GCA_004296435.1 | GSH8-2 | Laboratory of Bacterial Genomics, Pathogen Genomics Center, National Institute of Infectious Diseases  Wastewater treatment plant |
| GCF_004684305.1 | GCA_004684305.1 | WCX23 | Hunan Agricultural University |
| GCF_004684325.1 | GCA_004684325.1 | 23-C-23 | Hunan Agricultural University  Hunan, China |
| GCF_009791455.1 | GCA_009791455.1 | HX-3 | Ningbo University  Zhoushan, China |
| GCF_011602425.1 | GCA_011602425.1 | LHW39 | Huazhong Agricultural University  Hubei, China |
| GCF_012273595.1 | GCA_012273595.1 | NEB724 | NA |
| GCF_014161955.1 | GCA_014161955.1 | WP7-S18-ESBL-06 | Laboratory of Bacterial Genomics, Pathogen Genomics Center, National Institute of Infectious Diseases  Wastewater treatment plant |
| GCF_014169715.1 | GCA_014169715.1 | WP8-S18-ESBL-02 | Laboratory of Bacterial Genomics, Pathogen Genomics Center, National Institute of Infectious Diseases  Wastewater treatment plant |
| GCF_014679585.1 | GCA_014679585.1 | KAM330 | AMR Research Center, National Institute of Infectious Diseases |
| GCF_016026875.1 | GCA_016026875.1 | FDAARGOS_916 | FDA-ARGOS Database  Clinical or host-associated sample from *Aeromonas hydrophila* |
| GCF_016592295.1 | GCA_016592295.1 | RIMD111065 | Veterinary Public Health Veterinary Medicine, Kitasato University |
| GCF_017310195.1 | GCA_017310195.1 | Brac6 | MIGS Cultured Bacterial/Archaeal sample from Aeromonas hydrophila  Caatinga Biome |
| GCF_017310215.1 | GCA_017310215.1 | OnP3.1 | MIGS Cultured Bacterial/Archaeal sample from Aeromonas hydrophila  Caatinga Biome |
| GCF_018802385.1 | GCA_018802385.1 | 3019 | USDA  Pathogen: environmental/food/other sample from *Aeromonas hydrophila* |

**Table 3.** Identification of 02 *O. niloticus* complete sequence accessed through NCBI for vaccinology analysis.

| **Reference Sequence** | **Submitted Sequence** | **WGS project** | **Submitter** |
| --- | --- | --- | --- |
| GCF_000188235.2 | GCA_000188235.2 | AERX01 | Broad Institute |
| GCF_001858045.2 | GCA_001858045.3 | MKQE02 | University of Maryland |

**Table 4**. General information of the complete genomes of *Aeromonas hydrophila* from Vale do São Francisco

| **GenBank No.** | **Isolate Name** | **Genome Size** | **Number of Contigs** | **%GC** | **Total Genes** | **Coding genes** | **Non-coding genes** |
| --- | --- | --- | --- | --- | --- | --- | --- |
| GCA_017310195.1 | Brac6 | 4.8 Mb | One | 61.5 | 4,371 | 4,191 | 180 |
| GCA_019348925.1 | Aer_Brac14 | 5 Mb | 39 | 61 | 4,601 | 4,409 | 192 |
| GCA_022700855.1 | Aer_Brac14A | 4.8 Mb | One | 61.5 | 4,420 | 4,216 | 204 |
| GCA_019399935.1 | Aer_Brac46 | 4.9 Mb | 49 | 61.5 | 4,550 | 4,349 | 201 |
| GCA_019399965.1 | Brac54 | 4.7 Mb | 23 | 61.5 | 4,396 | 4,225 | 171 |
| GCA_022700815.1 | Aer_Brac66 | 4.8 Mb | One | 61.5 | 4,414 | 4,218 | 196 |
| GCA_017310135.1 | Aer_LaG33 | 4.8 Mb | 5 | 61.5 | 4,379 | 4,204 | 175 |
| GCA_017310075.1 | Aer_LaG34 | 4.7 Mb | 12 | 61.5 | 4,324 | 4,190 | 134 |
| GCA_018094775.1 | LaG38 | 4.7 Mb | 13 | 61.5 | 4,384 | 4,216 | 168 |
| GCA_019348795.1 | Aer_On1M | 5 Mb | 135 | 61 | 4,665 | 4,467 | 198 |
| GCA_019348875.1 | Aer_On3M | 5.2 Mb | 78 | 61 | 4,982 | 4,737 | 245 |
| GCA_038524185.1 | Aer_On7M | 5 Mb | 29 | 61 | 4,615 | 4,409 | 206 |
| GCA_018094725.1 | On9M | 4.9 Mb | 11 | 61 | 4,588 | 4,410 | 178 |
| GCA_021735805.1 | Ah_On10M | 5.1 Mb | 58 | 61 | 4,828 | 4,613 | 215 |
| GCA_053543895.1 | Aer_On11M | 5 Mb | 41 | 61 | 4,653 | 4,488 | 165 |
| GCA_053543875.1 | Aer_On16M | 5 Mb | 42 | 61 | 4,651 | 4,491 | 160 |
| GCA_053543855.1 | Aer_On18M | 4.9 Mb | 50 | 61 | 4,552 | 4,408 | 144 |
| GCA_019348745.1 | Aer_On19M | 4.8 Mb | 49 | 61.5 | 4,410 | 4,212 | 198 |
| GCA_053346735.1 | Aer_On21M | 4.9 Mb | 48 | 61 | 4,570 | 4,417 | 153 |
| GCA_019348935.1 | Aer_On22M | 5 Mb | 60 | 61 | 4,707 | 4,493 | 214 |
| GCA_021735825.1 | Ah_On23M | 5.3 Mb | 56 | 61 | 4,956 | 4,727 | 229 |
| GCA_019348915.1 | Aer_On27M | 4.9 Mb | 31 | 61 | 4,601 | 4,409 | 192 |
| GCA_016805405.1 | Aer_On29M | 4.8 Mb | 52 | 61.5 | 4,413 | 4,219 | 194 |
| GCA_019348835.1 | Aer_OnAA | 4.7 Mb | 20 | 61.5 | 4,376 | 4,208 | 168 |
| GCA_017310155.1 | Aer_OnP2.2 | 4.7 Mb | 13 | 61.5 | 4,372 | 4,203 | 169 |
| GCA_018094805.1 | OnP22 | 4.8 Mb | 10 | 61.5 | 4,405 | 4,236 | 169 |
| GCA_017310215.1 | OnP3.1 | 4.8 Mb | One | 61.5 | 4,370 | 4,191 | 179 |
| GCA_017310115.1 | Aer_OnP4.2 | 4.7 Mb | 4 | 61.5 | 4,371 | 4,201 | 170 |
| GCA_019348895.1 | Aer_OnRU3 | 5.2 Mb | 71 | 61 | 5,015 | 4,763 | 252 |
| GCA_019348855.1 | Aer_OnRU2 | 5.2 Mb | 56 | 61 | 5,004 | 4,761 | 243 |
| GCA_019399995.1 | Pi11 | 4.8 Mb | 41 | 61.5 | 4,432 | 4,249 | 183 |

Mb - megabase

**Table 5 -** General information on the complete genomes of *A.* *hyrophila* obtained from GenBank/NCBI

| **GenBank No.** | **Genome Size** | **Number of Contigs** | **%GC** | **Total Genes** | **Coding genes** | **Non-coding genes** |
| --- | --- | --- | --- | --- | --- | --- |
| GCA_000940915.1 | 4.9 Mb | 4 | 61.0 | 4,587 | 4,382 | 152 |
| GCA_000963645.1 | 4.9 Mb | 1 | 61.0 | 4,541 | 4,319 | 165 |
| GCA_001455365.1 | 5.1Mb | 4 | 60.5 | 4,812 | 4,578 | 168 |
| GCA_001518775.1 | 5.3 Mb | 5 | 60.0 | 4,930 | 4,702 | 156 |
| GCF_001683535.1 | 5.0 Mb | 1 | 60.5 | 4,549 | 4,337 | 154 |
| GCF_001687125.1 | 5.0 Mb | two | 61.0 | 4,731 | 4,497 | 162 |
| GCF_001895965.1 | 4.8Mb | 1 | 61.5 | 4,414 | 4,201 | 162 |
| GCF_003014775.1 | 4.9 Mb | 1 | 61.0 | 4,603 | 4,386 | 165 |
| GCF_003491225.1 | 5.2Mb | 1 | 60.5 | 4,772 | 4,540 | 162 |
| GCF_003491245.1 | 5.0 Mb | 1 | 61.0 | 4,565 | 4,361 | 162 |
| GCF_004296435.1 | 5.0 Mb | two | 61.0 | 4,601 | 4,375 | 161 |
| GCF_004684305.1 | 5.0 Mb | two | 60.0 | 4,990 | 4,726 | 165 |
| GCF_004684325.1 | 5.3Mb | two | 60.5 | 4,948 | 4,687 | 165 |
| GCF_009791455.1 | 4.9 Mb | 1 | 61.0 | 4,523 | 4,313 | 163 |
| GCF_011602425.1 | 5.1Mb | 1 | 60.5 | 4,722 | 4,506 | 146 |
| GCF_012273595.1 | 4.6Mb | 1 | 61.5 | 4,238 | 3,946 | 161 |
| GCF_014161955.1 | 4.9 Mb | 1 | 61.0 | 4534 | 4,314 | 162 |
| GCF_014169715.1 | 5.0 Mb | two | 61.0 | 4,601 | 4,375 | 161 |
| GCF_014679585.1 | 5.4Mb | 10 | 60.5 | 5,134 | 4,860 | 165 |
| GCF_016026875.1 | 4.7Mb | 1 | 61.5 | 4,387 | 4,192 | 160 |
| GCF_016592295.1 | 5.0 Mb | 1 | 61.0 | 4,540 | 4,331 | 162 |
| GCF_017310195.1 | 4.8Mb | 1 | 61.0 | 4,372 | 4,195 | 151 |
| GCF_017310215.1 | 4.8Mb | 1 | 61.0 | 4,370 | 4,194 | 151 |
| GCF_018802385.1 | 5.1Mb | 1 | 60.5 | 4,847 | 4,565 | 168 |

Mb - megabase

**Table 6 -** General information on the complete genomes of *O. niloticus* obtained from GenBank/NCBI

| **GenBank No.** | **Genome Size** | **Number of Contigs** | **%GC** | **Total Genes** | **Coding genes** | **Non-coding genes** |
| --- | --- | --- | --- | --- | --- | --- |
| GCF_000188235.2 | 927.7 Mb | 77,754 | n/a | n/a | n/a | n/a |
| GCF_001858045.2 | 1Gb | 3,009 | 40.5 | 42,622 | 29,550 | 8882 |

Mb - megabase, Gb – Gigabase, n/a - Information not noted.

**Table 7**. Relation of 178 proteins considered vaccine targets by Vaxign, adhesion probability and size.

| **Protein Access** | **Adhesion probability** | **Protein size** |
| --- | --- | --- |
| Ah_Brac14_18570 | 0.216 | 623 |
| Ah_Brac14_24340 | 0.591 | 152 |
| Ah_Brac14_32510 | 0.327 | 132 |
| Ah_Brac14_44270 | 0.762 | 164 |
| Ah_Brac14_31990 | 0.180 | 403 |
| Ah_Brac14_32050 | 0.561 | 126 |
| Ah_Brac14_32060 | 0.159 | 366 |
| Ah_Brac14_32140 | 0.332 | 229 |
| Ah_Brac14_32150 | 0,150 | 133 |
| Ah_Brac14_32360 | 0.228 | 288 |
| Ah_Brac14_32620 | 0.296 | 259 |
| Ah_Brac14_32650 | 0.213 | 224 |
| Ah_Brac14_32730 | 0.209 | 532 |
| Ah_Brac14_42190 | 0.275 | 195 |
| Ah_Brac14_15110 | 0.222 | 154 |
| Ah_Brac14_15150 | 0.197 | 137 |
| Ah_Brac14_30190 | 0.540 | 149 |
| Ah_Brac14_35090 | 0.365 | 435 |
| Ah_Brac14_35380 | 0.250 | 229 |
| Ah_Brac14_36560 | 0.225 | 240 |
| Ah_Brac14_42160 | 0.809 | 805 |
| Ah_Brac14_42230 | 0.238 | 423 |
| Ah_Brac14_42240 | 0.224 | 308 |
| Ah_Brac14_00280 | 0.418 | 526 |
| Ah_Brac14_06970 | 0.219 | 431 |
| Ah_Brac14_07570 | 0.666 | 262 |
| Ah_Brac14_07660 | 0.726 | 280 |
| Ah_Brac14_07680 | 0.268 | 315 |
| Ah_Brac14_08380 | 0.410 | 206 |
| Ah_Brac14_08560 | 0.171 | 298 |
| Ah_Brac14_08580 | 0.644 | 481 |
| Ah_Brac14_08700 | 0.262 | 202 |
| Ah_Brac14_09570 | 0.794 | 284 |
| Ah_Brac14_09630 | 0.338 | 447 |
| Ah_Brac14_10700 | 0.719 | 399 |
| Ah_Brac14_10920 | 0.378 | 349 |
| Ah_Brac14_11130 | 0.331 | 455 |
| Ah_Brac14_11500 | 0.566 | 132 |
| Ah_Brac14_11510 | 0.693 | 158 |
| Ah_Brac14_11720 | 0.160 | 181 |
| Ah_Brac14_12230 | 0.591 | 561 |
| Ah_Brac14_12250 | 0.215 | 306 |
| Ah_Brac14_12550 | 0.383 | 227 |
| Ah_Brac14_13320 | 0.679 | 286 |
| Ah_Brac14_13430 | 0.343 | 132 |
| Ah_Brac14_13880 | 0.294 | 332 |
| Ah_Brac14_14150 | 0.521 | 453 |
| Ah_Brac14_14550 | 0.340 | 313 |
| Ah_Brac14_14590 | 0.175 | 164 |
| Ah_Brac14_24490 | 0.401 | 276 |
| Ah_Brac14_26960 | 0.236 | 173 |
| Ah_Brac14_30390 | 0.409 | 278 |
| Ah_Brac14_30460 | 0.230 | 151 |
| Ah_Brac14_30610 | 0.241 | 259 |
| Ah_Brac14_36510 | 0.477 | 106 |
| Ah_Brac14_40760 | 0.550 | 611 |
| Ah_Brac14_42680 | 0.287 | 398 |
| Ah_Brac14_01090 | 0.140 | 138 |
| Ah_Brac14_01270 | 0.318 | 358 |
| Ah_Brac14_01650 | 0.105 | 799 |
| Ah_Brac14_01720 | 0.336 | 230 |
| Ah_Brac14_01730 | 0.357 | 246 |
| Ah_Brac14_01890 | 0.425 | 296 |
| Ah_Brac14_02000 | 0.181 | 160 |
| Ah_Brac14_02200 | 0.288 | 523 |
| Ah_Brac14_02520 | 0.239 | 245 |
| Ah_Brac14_02530 | 0.765 | 186 |
| Ah_Brac14_03020 | 0.232 | 638 |
| Ah_Brac14_03130 | 0.295 | 224 |
| Ah_Brac14_03160 | 0.409 | 394 |
| Ah_Brac14_03200 | 0.422 | 367 |
| Ah_Brac14_03980 | 0.238 | 210 |
| Ah_Brac14_04000 | 0.596 | 176 |
| Ah_Brac14_04560 | 0.274 | 176 |
| Ah_Brac14_04600 | 0.183 | 246 |
| Ah_Brac14_06050 | 0.241 | 122 |
| Ah_Brac14_06150 | 0.569 | 350 |
| Ah_Brac14_06230 | 0.365 | 458 |
| Ah_Brac14_06310 | 0,309 | 104 |
| Ah_Brac14_06480 | 0.237 | 299 |
| Ah_Brac14_06610 | 0.220 | 56 |
| Ah_Brac14_06690 | 0.191 | 204 |
| Ah_Brac14_09270 | 0.432 | 98 |
| Ah_Brac14_10010 | 0.448 | 266 |
| Ah_Brac14_17990 | 0.323 | 262 |
| Ah_Brac14_18380 | 0.203 | 411 |
| Ah_Brac14_18720 | 0.155 | 156 |
| Ah_Brac14_18750 | 0.670 | 657 |
| Ah_Brac14_18760 | 0.206 | 338 |
| Ah_Brac14_18770 | 0.132 | 355 |
| Ah_Brac14_19030 | 0.548 | 282 |
| Ah_Brac14_19400 | 0.297 | 333 |
| Ah_Brac14_19650 | 0.066 | 343 |
| Ah_Brac14_19720 | 0.187 | 319 |
| Ah_Brac14_20340 | 0.530 | 145 |
| Ah_Brac14_20540 | 0.590 | 200 |
| Ah_Brac14_20660 | 0.552 | 137 |
| Ah_Brac14_20800 | 0.218 | 245 |
| Ah_Brac14_20830 | 0.286 | 224 |
| Ah_Brac14_20960 | 0.342 | 215 |
| Ah_Brac14_20970 | 0.374 | 216 |
| Ah_Brac14_21030 | 0.205 | 541 |
| Ah_Brac14_21040 | 0.332 | 335 |
| Ah_Brac14_21130 | 0.328 | 420 |
| Ah_Brac14_21490 | 0.271 | 298 |
| Ah_Brac14_21710 | 0.257 | 423 |
| Ah_Brac14_21810 | 0.207 | 209 |
| Ah_Brac14_21830 | 0.156 | 183 |
| Ah_Brac14_21840 | 0.278 | 143 |
| Ah_Brac14_22050 | 0.273 | 640 |
| Ah_Brac14_22110 | 0.298 | 172 |
| Ah_Brac14_22320 | 0.805 | 341 |
| Ah_Brac14_22440 | 0.292 | 247 |
| Ah_Brac14_22530 | 0.499 | 193 |
| Ah_Brac14_22570 | 0.160 | 81 |
| Ah_Brac14_22950 | 0.439 | 680 |
| Ah_Brac14_22970 | 0.178 | 345 |
| Ah_Brac14_23040 | 0.411 | 212 |
| Ah_Brac14_23320 | 0.235 | 281 |
| Ah_Brac14_23430 | 0.343 | 238 |
| Ah_Brac14_23600 | 0.480 | 155 |
| Ah_Brac14_23670 | 0.090 | 406 |
| Ah_Brac14_23790 | 0.232 | 365 |
| Ah_Brac14_23990 | 0.194 | 53 |
| Ah_Brac14_24200 | 0.360 | 128 |
| Ah_Brac14_24960 | 0.173 | 252 |
| Ah_Brac14_25230 | 0.175 | 303 |
| Ah_Brac14_25270 | 0.324 | 408 |
| Ah_Brac14_25430 | 0.3359 | 304 |
| Ah_Brac14_25840 | 0.813 | 355 |
| Ah_Brac14_25920 | 0.209 | 274 |
| Ah_Brac14_25940 | 0.225 | 149 |
| Ah_Brac14_26180 | 0.273 | 543 |
| Ah_Brac14_26720 | 0.261 | 653 |
| Ah_Brac14_26740 | 0.307 | 230 |
| Ah_Brac14_27160 | 0.166 | 157 |
| Ah_Brac14_22320 | 0.805 | 341 |
| Ah_Brac14_27210 | 0.782 | 187 |
| Ah_Brac14_27390 | 0.423 | 259 |
| Ah_Brac14_27440 | 0.316 | 239 |
| Ah_Brac14_28290 | 0.598 | 160 |
| Ah_Brac14_28330 | 0.000 | 30 |
| Ah_Brac14_28730 | 0.412 | 328 |
| Ah_Brac14_28810 | 0.203 | 271 |
| Ah_Brac14_28940 | 0.511 | 270 |
| Ah_Brac14_28960 | 0.255 | 474 |
| Ah_Brac14_29000 | 0.387 | 224 |
| Ah_Brac14_29090 | 0.201 | 280 |
| Ah_Brac14_29100 | 0.143 | 301 |
| Ah_Brac14_31070 | 0.185 | 393 |
| Ah_Brac14_31680 | 0.482 | 316 |
| Ah_Brac14_31700 | 0.421 | 476 |
| Ah_Brac14_31770 | 0.289 | 277 |
| Ah_Brac14_31840 | 0.381 | 69 |
| Ah_Brac14_35940 | 0.274 | 308 |
| Ah_Brac14_36210 | 0.340 | 415 |
| Ah_Brac14_36250 | 0.603 | 180 |
| Ah_Brac14_36340 | 0.398 | 257 |
| Ah_Brac14_36690 | 0.204 | 442 |
| Ah_Brac14_37200 | 0.413 | 290 |
| Ah_Brac14_37460 | 0.286 | 480 |
| Ah_Brac14_37580 | 0.147 | 96 |
| Ah_Brac14_37650 | 0.243 | 206 |
| Ah_Brac14_37670 | 0.347 | 224 |
| Ah_Brac14_37820 | 0.323 | 371 |
| Ah_Brac14_37920 | 0.400 | 481 |
| Ah_Brac14_38670 | 0.349 | 245 |
| Ah_Brac14_38680 | 0.487 | 225 |
| Ah_Brac14_39890 | 0.606 | 702 |
| Ah_Brac14_39990 | 0.244 | 221 |
| Ah_Brac14_41790 | 0.704 | 363 |
| Ah_Brac14_41800 | 0.365 | 439 |
| Ah_Brac14_41860 | 0.296 | 165 |
| Ah_Brac14_42700 | 0.421 | 383 |
| Ah_Brac14_42810 | 0.254 | 252 |
| Ah_Brac14_43110 | 0.222 | 299 |
| Ah_Brac14_43120 | 0.248 | 258 |
| Ah_Brac14_43160 | 0.282 | 146 |
| Ah_Brac14_44540 | 0.175 | 421 |

Fonte: Autor, 2022.

**Table 8 -** Result of the analysis of proteins predicted as the best vaccine targets by VAXIGN/DEG

| **Protein Accession** | **Location** | **Probability*** | **Adhesin Probability** | **Trans-member helices** | **Protein Length** |
| --- | --- | --- | --- | --- | --- |
| Ah_Brac14_25840 | Outer Membrane | 0.949 | 0.813 | 1 | 355 |
| Ah_Brac14_42160 | Extracellular | 1 | 0.809 | 0 | 805 |
| Ah_Brac14_22320 | Extracellular | 0.971 | 0.805 | 0 | 341 |
| Ah_Brac14_09570 | Unknown | 0.658 | 0.794 | 1 | 284 |
| Ah_Brac14_27210 | Unknown | 0.25 | 0.782 | 0 | 187 |
| Ah_Brac14_02530 | Unknown | 0.25 | 0.765 | 0 | 186 |
| Ah_Brac14_44270 | Unknown | 0.25 | 0.762 | 0 | 164 |
| Ah_Brac14_07660 | Outer Membrane | 0.993 | 0.726 | 0 | 280 |
| Ah_Brac14_10700 | Unknown | 0.496 | 0.719 | 0 | 399 |
| Ah_Brac14_41790 | Outer Membrane | 1 | 0.704 | 0 | 363 |
| Ah_Brac14_11510 | Unknown | 0.25 | 0.693 | 0 | 158 |
| Ah_Brac14_13320 | Unknown | 0.25 | 0.679 | 0 | 286 |
| Ah_Brac14_18750 | Outer Membrane | 1 | 0.670 | 0 | 657 |
| Ah_Brac14_07570 | Unknown | 0.2 | 0.666 | 1 | 262 |
| Ah_Brac14_08580 | Unknown | 0.25 | 0.644 | 0 | 481 |
| Ah_Brac14_39890 | Outer Membrane | 1 | 0.606 | 0 | 702 |
| Ah_Brac14_36250 | Unknown | 0.25 | 0.603 | 0 | 180 |
| Ah_Brac14_28290 | Cytoplasmic Membrane | 1 | 0.598 | 4 | 160 |
| Ah_Brac14_04000 | Unknown | 0.2 | 0.596 | two | 176 |
| Ah_Brac14_24340 | Unknown | 0.25 | 0.591 | 0 | 152 |
| Ah_Brac14_12230 | Periplasmic | 1 | 0.591 | 0 | 561 |
| Ah_Brac14_20540 | Unknown | 0.2 | 0.590 | 0 | 200 |
| Ah_Brac14_06150 | Outer Membrane | 0.993 | 0.569 | 0 | 350 |
| Ah_Brac14_11500 | Unknown | 0.25 | 0.566 | 0 | 132 |
| Ah_Brac14_32050 | Unknown | 0.25 | 0.561 | 0 | 126 |
| Ah_Brac14_20660 | Cytoplasmic Membrane | 0.982 | 0.552 | 1 | 137 |
| Ah_Brac14_40760 | Outer Membrane | 1 | 0.550 | 0 | 611 |
| Ah_Brac14_19030 | Cytoplasmic Membrane | 1 | 0.548 | 6 | 282 |
| Ah_Brac14_30190 | Periplasmic | 0.984 | 0.540 | 0 | 149 |
| Ah_Brac14_20340 | Cytoplasmic Membrane | 1 | 0.530 | 4 | 145 |
| Ah_Brac14_14150 | Unknown | 0.25 | 0.521 | 0 | 453 |
| Ah_Brac14_28940 | Cytoplasmic Membrane | 1 | 0.511 | 6 | 270 |

***Probability:** the protein location probability of being right, it varies from 0.0 to 1.0.

| **query_id** | **subject_id** | **pct_identity** | **aln_length** | **n_of_mismatches** | **gap_openings** | **q_start** | **q_end** | **s_start** | **s_end** | **e_value** | **bit_score** |
| --- | --- | --- | --- | --- | --- | --- | --- | --- | --- | --- | --- |
| Ah_Brac14_25840 | [DEG10270178](http://origin.tubic.org/deg/public/index.php/information/bacteria/deg10270178.html) | 36.62 | 355 | 204 | 9 | 19 | 355 | 30 | 381 | 1.59E-62 | 204 |
| Ah_Brac14_09570 | [DEG10520166](http://origin.tubic.org/deg/public/index.php/information/bacteria/deg10520166.html) | 34.483 | 145 | 69 | 4 | 160 | 278 | 482 | 626 | 3.30E-11 | 62.8 |
| Ah_Brac14_02530 | [DEG10030691](http://origin.tubic.org/deg/public/index.php/information/bacteria/deg10030691.html) | 44.845 | 194 | 92 | 6 | 3 | 186 | 1 | 189 | 3.27E-46 | 149 |
| Ah_Brac14_10700 | [DEG10350479](http://origin.tubic.org/deg/public/index.php/information/bacteria/deg10350479.html) | 32.71 | 107 | 67 | 4 | 291 | 395 | 406 | 509 | 9.34E-06 | 47 |
| Ah_Brac14_41790 | [DEG10260047](http://origin.tubic.org/deg/public/index.php/information/bacteria/deg10260047.html) | 38.402 | 388 | 204 | 12 | 1 | 363 | 1 | 378 | 1.95E-62 | 203 |
| Ah_Brac14_13320 | [DEG10350339](http://origin.tubic.org/deg/public/index.php/information/bacteria/deg10350339.html) | 50.185 | 271 | 128 | 2 | 23 | 286 | 29 | 299 | 1.47E-90 | 274 |
| Ah_Brac14_18750 | [DEG10130215](http://origin.tubic.org/deg/public/index.php/information/bacteria/deg10130215.html) | 30.653 | 199 | 122 | 6 | 3 | 187 | 10 | 206 | 2.15E-18 | 89 |
| Ah_Brac14_39890 | [DEG10130224](http://origin.tubic.org/deg/public/index.php/information/bacteria/deg10130224.html) | 20.644 | 683 | 474 | 21 | 61 | 702 | 69 | 724 | 7.78E-19 | 90.5 |
| Ah_Brac14_36250 | [DEG10050022](http://origin.tubic.org/deg/public/index.php/information/bacteria/deg10050022.html) | 46.927 | 179 | 92 | 1 | 1 | 176 | 1 | 179 | 1.55E-49 | 157 |
| Ah_Brac14_28290 | [DEG10180172](http://origin.tubic.org/deg/public/index.php/information/bacteria/deg10180172.html) | 55.629 | 151 | 65 | 2 | 1 | 149 | 1 | 151 | 1.44E-51 | 161 |
| Ah_Brac14_04000 | [DEG10080004](http://origin.tubic.org/deg/public/index.php/information/bacteria/deg10080004.html) | 35.802 | 162 | 85 | 6 | 10 | 159 | 6 | 160 | 2.03E-26 | 98.2 |
| Ah_Brac14_12230 | [DEG10450345](http://origin.tubic.org/deg/public/index.php/information/bacteria/deg10450345.html) | 25.752 | 532 | 319 | 23 | 3 | 518 | 10 | 481 | 1.26E-20 | 94.7 |
| Ah_Brac14_06150 | [DEG10260013](http://origin.tubic.org/deg/public/index.php/information/bacteria/deg10260013.html) | 40.625 | 352 | 168 | 7 | 10 | 339 | 10 | 342 | 2.26E-66 | 212 |
| Ah_Brac14_32050 | [DEG10560354](http://origin.tubic.org/deg/public/index.php/information/bacteria/deg10560354.html) | 36.667 | 90 | 57 | 0 | 23 | 112 | 41 | 130 | 1.92E-14 | 64.3 |
| Ah_Brac14_40760 | [DEG10130215](http://origin.tubic.org/deg/public/index.php/information/bacteria/deg10130215.html) | 26.906 | 223 | 137 | 7 | 26 | 228 | 44 | 260 | 1.45E-10 | 63.5 |
| Ah_Brac14_19030 | [DEG10050083](http://origin.tubic.org/deg/public/index.php/information/bacteria/deg10050083.html) | 52.574 | 272 | 129 | 0 | 10 | 281 | 13 | 284 | 3.26E-103 | 301 |
| Ah_Brac14_30190 | [DEG10350120](http://origin.tubic.org/deg/public/index.php/information/bacteria/deg10350120.html) | 54.43 | 79 | 36 | 0 | 70 | 148 | 84 | 162 | 2.23E-25 | 94.4 |
| Ah_Brac14_28940 | [DEG10050083](http://origin.tubic.org/deg/public/index.php/information/bacteria/deg10050083.html) | 32.093 | 215 | 122 | 6 | 56 | 248 | 69 | 281 | 2.83E-17 | 79 |

**Table 9.** Results of 18 best proteins by DEG (Database of essential genes)

**Table 10.** List of the 10 best vaccine candidates identified by analyzes of the Vaxigin and DEG programs.

| **Protein** | **Protein ID** | | **Locus tag** | **Gene Name** | **Domain (Interpro)** | | **Product** | **Subcellular Location**  **PSORTb/Surfg** | | | **TMHMM** | **Signal-P** | | **Probability Signal Peptide** | | **Probability of adherence** | **Length (AA)** | | **Mass (KDa)** |
| --- | --- | --- | --- | --- | --- | --- | --- | --- | --- | --- | --- | --- | --- | --- | --- | --- | --- | --- | --- |
| **Ah_Brac14_**  **25840** | | WP_219313282.1 | WP_219313282 | HFP99_04160 | AttH domain - Beta barrel domain | carotenoid 1,2-hydratase | | | Outer membrane/SEC | 1 | | | Yes (between 22-23) | 0.8374 | 0.813 | | 355 | 43.845 | |
| **Ah_Brac14_**  **09570** | | WP_219311764.1 | WP_219311764 | FT673_17865 | Soluble lytic murine transglycosylase L domain | Lytic transglycosylase | | | Unknow/SEC | 1 | | | Yes (between 27-28) | 0.5664 | 0.794 | | 284 | 30.995 | |
| **Ah_Brac14_**  **02530** | | WP_011705502.1 | WP_011705502 | HQK30_07795 | Lipid/polyisoprenoid-bd_YceI | YceI family proteins | | | Unknow/SEC | 0 | | | Yes (between 20-21) | 0.9296 | 0.765 | | 186 | 20.293 | |
| **Ah_Brac14_**  **10700** | | WP_219311898.1 | WP_219311898 | ccmE | Cytochrome c-like domain | Cytochrome c-type biogenesis protein CcmE | | | Unknow/SEC | 0 | | | Yes (between 23-24) | 0.3470 | 0.719 | | 399 | 42.907 | |
| **Ah_Brac14_**  **41790** | | WP_113995956.1 | WP_113995956 | lamB | Porin domain superfamily, Gram-negative type | Maltoporin | | | Outer membrane/SEC | 0 | | | Yes (between 21-22) | 0.9945 | 0.704 | | 357 | 38.704 | |
| **Ah_Brac14_**  **13320** | | WP_219312142.1 | WP_219312142 | HQK30_07840 | Lactamase_B | MBL fold metallohydrolase | | | Unknow/SEC | 0 | | | Yes (between 22-23) | 0.9648 | 0.679 | | 286 | 30.968 | |
| **Ah_Brac14_**  **18750** | | WP_219312629.1 | WP_219312629 | btuB | Plug/TonB_dep_Rec | Vitamin B12 BtuB transporter | | | Outer membrane/SEC | 0 | | | Yes (between 28-29) | 0.7598 | 0.670 | | 657 | 71.661 | |
| **Ah_Brac14_**  **39890** | | WP_029305116.1 | WP_029305116 | AHA_0461 | TonB-dependent receptor, plug domain/TonB-dependent receptor-like, beta-barrel | TonB-dependent siderophore receptor | | | Outer membrane/SEC | 0 | | | Yes (between 34-35) | 0.9597 | 0.606 | | 709 | 78.279 | |
| **Ah_Brac14_**  **36250** | | [WP_011707296.1](https://www.ncbi.nlm.nih.gov/protein/WP_043168099.1?report=genbank&log$=protalign&blast_rank=2&RID=22P8UVVT016) | WP_011707296 | AHGSH82_  007470 | Conserved hypothetical protein CHP01626, YtfJ | YtfJ family proteins | | | Unknow/SEC | 0 | | | Yes (between 17-18) | 0.9642 | 0.603 | | 180 | 19.61 | |
| **Ah_Brac14_**  **28290** | WP_043160143.1 | | WP_043160143 | AHGSH82_  002220 | Protein of unknown function DUF1097 | | DUF1097 domain-containing protein | Cytoplasmic Membrane/MEM | | | 4 | No | | ___ | | 0.598 | 160 | | 16.736 |

Caption: AA – amino acids; KDa – Kilodalton; PSE - Potentially Surface-Exposed; SEC - secreted; MEM – membrane.
